# STDs help line and prevention

**DOI:** 10.1186/1742-4690-9-S1-P122

**Published:** 2012-05-25

**Authors:** Filippo Maria Taglieri, Pietro Gallo, Anna Colucci, Anna Maria Luzi, Rudi Valli, Francesca Botta, Eleonora Lichtner

**Affiliations:** 1 I.S.S., Rome, Italy

## Introduction

Sexually Transmitted Diseases (STDs) are a public health problem worldwide, each year about 340 million people, aged between 15 and 49, who contract an STDs (WHO). The Research Unit psycho-socio-behavioral, Communication, Training – U.O. RCF (Department of Infectious Diseases, Parasitic and Immunomediated – National Institute of Health in Italy ) initiated in 2010 under a project funded by the Ministry of Health, a specific STDs prevention activities through the intervention of telephone counseling. This action is already used by researchers at the U.O. RCF since 1987 for the prevention of HIV infection and AIDS.

## Materials and methods

The data collected, anonymously, during the telephone counseling intervention were entered and stored in a data-entry software that has allowed, through statistical analysis, to define some personal characteristics of those users who choose the telephone to get information on STDs, and to identify their information needs.

## Results

In the period 21 June 2010 - June 20, 2011 U.O. RCF received 2,017 phone calls during which it as focused attention on STDs. The 91.1% of the people-users are male, 77.3% were on no more than 39 years. The greatest number of calls coming from the North Italy (46%), followed by the Centre (30%), South (18%) and the Islands (6%). Data analysis can distinguish three main groups of people-people: heterosexual, homosexual-bisexual and people with no risk factor. The information area of interest for people-users about the method of transmission of infectious agents of STDs (60%) (figure 1).

## Conclusions

These data would seem to suggest the need for interventions aimed at prevention of STDs populations of youth, as well as information strategies able to reach and engage the female target. The value added of telephone counseling for STDs is to provide the person-user cognitive tools, to help you avoid risky behavior and enable empowerment processes aimed at protecting the health of the individual and his community.

